# T4 Pili Promote Colonization and Immune Evasion Phenotypes of Nonencapsulated M4 Streptococcus pyogenes

**DOI:** 10.1128/mBio.01580-20

**Published:** 2020-07-21

**Authors:** Yi-Hsuan Chen, Shao-Hui Li, Yao-Cheng Yang, Shu-Hao Hsu, Victor Nizet, Yung-Chi Chang

**Affiliations:** a Graduate Institute of Microbiology, College of Medicine, National Taiwan University, Taipei, Taiwan; b Graduate Institute of Anatomy and Cell Biology, College of Medicine, National Taiwan University, Taipei, Taiwan; c Department of Pediatrics, University of California, San Diego, La Jolla, California, USA; d Skaggs School of Pharmacy and Pharmaceutical Sciences, University of California, San Diego, La Jolla, California, USA; KUMC

**Keywords:** *Streptococcus pyogenes*, group A *Streptococcus*, pilus, T antigen, haptoglobin, innate immunity, virulence factor

## Abstract

Streptococcus pyogenes (group A *Streptococcus* [GAS]) is an important human pathogen causing a broad spectrum of diseases and associated with significant global morbidity and mortality. Almost all GAS isolates express a surface hyaluronic acid capsule, a virulence determinant that facilitates host colonization and impedes phagocyte killing. However, recent epidemiologic surveillance has reported a sustained increase in both mucosal and invasive infections caused by nonencapsulated GAS, which questions the indispensable role of hyaluronic acid capsule in GAS pathogenesis. In this study, we found that pilus of M4 GAS not only significantly promotes biofilm formation, adherence, and cytotoxicity to human upper respiratory tract epithelial cells and keratinocytes, but also promotes survival in human whole blood and increased virulence in murine models of invasive infection. T4 antigen, the pilus backbone protein of M4 GAS, binds haptoglobin, an abundant human acute-phase protein upregulated upon infection and inflammation, on the bacterial surface. Haptoglobin sequestration reduces the susceptibility of nonencapsulated M4 GAS to antimicrobial peptides released from activated neutrophils and platelets. Our results reveal a previously unappreciated virulence-promoting role of M4 GAS pili, in part mediated by co-opting the biology of haptoglobin to mitigate host antimicrobial defenses.

## Abstract (precis)

Group A *Streptococcus* (GAS) is a strict human pathogen causing more than 700 million infections globally each year. The majority of the disease-causing GAS are encapsulated, which greatly guarantees survival and dissemination in the host. Emergence of the capsule-negative GAS, such as M4 GAS, in recent epidemiologic surveillance alarms the necessity to elucidate the virulence determinants of these pathogens. Here, we found that M4 pili play an important role in promoting M4 GAS adherence and cytotoxicity to human pharyngeal epithelial cells and keratinocytes. The same molecule also significantly enhanced M4 GAS survival and replication in human whole blood and experimental murine infection. T4 antigen, which composes the backbone of M4 pili, was able to sequester the very abundant serum protein haptoglobin to further confer M4 GAS resistance to antibacterial substances released by neutrophils and platelets.

## INTRODUCTION

Streptococcus pyogenes (also known as group A *Streptococcus* [GAS]) is an exclusively human pathogen that causes more than 700 million infections globally each year (1). The resulting diseases range from mild infections of the throat and skin to devastating invasive infections, such as streptococcal toxic shock syndrome and necrotizing fasciitis to poststreptococcal immune-mediated sequelae of acute rheumatic fever, rheumatic heart disease, and glomerulonephritis (2–4). The hyaluronic acid (HA) capsule, a major virulence factor expressed by the vast majority of GAS strains for full pathogenesis, has antiphagocytic, adhesive, and signaling properties that cooperatively promote colonization, subvert host antibacterial responses, and contribute to invasive disease potential (5–8). However, recent epidemiologic surveillance has reported a sustained increase in both mucosal and invasive infections caused by nonencapsulated GAS, including all tested isolates of the M4 and M22 serotypes (9–11) and some recent emerging isolates of M28, M87, and M89 serotypes (12–14). These observations indicate that HA capsule expression is somehow dispensable for GAS disease pathogenesis, provided the strains are equipped with alternative virulence-related mechanisms to interact with the host and thwart the host immune responses to survive and spread *in vivo*.

M4 GAS is one of the major serotypes identified in mucosal and invasive GAS infections despite lacking the entire *hasABC* operon encoding HA capsule biosynthesis (10, 11, 15–18). Unlike experimentally derived capsule-deficient mutants from encapsulated GAS serotype strains that showed extremely attenuated virulence in infected mice, M4 GAS clinical isolates replicated efficiently in human whole blood and caused invasive infection in experimental animals (6, 10, 18, 19). In addition, heterologous expression of the HA capsule operon in an M4 GAS isolate did not enhance the strain’s survival in human blood or its *in vivo* virulence in mice (18). Expression of fibronectin-binding protein (Fba) and the host complement regulator C4b binding protein (C4BP) on the bacterial surface was suggested to endow M4 GAS with host cell adherence and phagocyte resistance properties, respectively (18, 20); however, the detailed molecular mechanisms that confer full virulence potential to nonencapsulated M4 GAS strains remain largely unexplored.

Pili, long filamentous structures extending from the bacterial surface, are multifunctional GAS virulence determinants involved in host colonization, biofilm formation, and modulation of host antibacterial immune responses in a manner dependent on pilus type (21–26). Fibronectin-binding, collagen-binding T antigen (FCT) regions are GAS pilus genetic loci composed of genes encoding backbone proteins (also known as the Lancefield T antigen), accessory proteins, pilus-associated sortases and transcriptional regulators (27–29). Nine different FCT regions have been identiﬁed in GAS based on the gene organization and sequence variation of the *tee* gene encoding the T antigen (29–31). The biological function of T antigens among GAS of difference pilus types remains to be elucidated.

GAS strains interact with a variety of host serum factors, including fibrinogen, fibronectin, immunoglobulins, plasminogen, factor H, and C4BP, and these binding activities play important roles during colonization and the infectious process (32, 33). A specific interaction between T4 antigen-carrying GAS and haptoglobin (Hp) has been documented since the 1970s, but without a defined biological function attributed to it (34, 35). Hp is an abundant acute-phase protein produced by hepatocytes upon infection and various environmental insults which has been shown to bind to human neutrophils and monocytes and inhibit their respiratory burst, chemotaxis, phagocytosis, inflammation, and bactericidal activities (36–40). These published data led us to hypothesize that T4 antigen of M4 GAS could co-opt Hp to exploit its immune-suppressive properties and interfere with host defense mechanisms.

To test our hypothesis, the *spy0116* gene that encodes the pilus backbone protein (T4 antigen) was eliminated in the M4 GAS background by precise allelic replacement mutagenesis to elucidate its contribution to host colonization and antimicrobial immune responses. We find that T4 antigen plays an important role in promoting M4 GAS adherence and cytotoxicity to human pharyngeal epithelial cells and keratinocytes while also enhancing M4 GAS survival and replication in human whole blood and experimental murine infection. Finally, surface acquisition of Hp by T4 antigen confers M4 GAS resistance to antibacterial substances released by neutrophils and platelets.

## RESULTS

### T4 antigen backbone pilin is essential for FCT-5-type pilus formation in M4 GAS.

**FIG 1:**
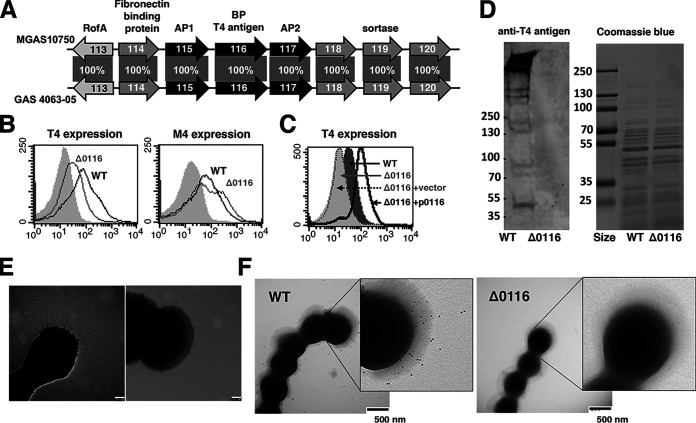
Deletion of the *spy0116* gene in M4 GAS FCT-5 region abrogated pilus expression. (A) Schematic representation of the FCT-5 pilus genes and pilus-assembly sortases of M4 GAS strain 4063-05 with a comparison to the M4 reference strain MGAS10750. Nucleotide sequence identity between strains is shown in percentages. (B) Analysis of surface T4 (Spy0116) and M4 expression in M4 GAS and the Δ0116 mutant by flow cytometry with antibodies recognizing T4 antigen and M4 protein, respectively. (C) Surface T4 expression of the Δ0116+p0116 complemented strain. (D) Western blot analysis of pilus expression in M4 GAS and the Δ0116 mutant using anti-T4 antigen antibodies. Transmission electron microscopic analysis of M4 GAS pili with phosphotungstic acid negative staining (E) and immunogold labeling using anti-T4 antigen antibodies (F).

Whole-genome sequencing was first applied to M4 strain 4063-05 to obtain the draft genome and the nucleotide sequence of the FCT region (see Fig. S1A in the supplemental material). The FCT region of strain 4063-05 is ∼14.5 kb in length, with 100% nucleotide sequence identify to the M4 GAS reference strain MGAS10750. Eight complete open reading frames are evident, comprising genes encoding a regulator of transcription (*spy0113*), four cell wall-anchored surface proteins (*spy0114* to *spy0117*), and three putative sortases (*spy0118* to *spy120*) (Fig. 1A). As prior studies of GAS pili centered on a few pilus types, and because biological functions attributed to the T4 antigen and FCT-5-type pilus of M4 GAS have not previously been explored, we then performed precise allelic exchange mutagenesis to delete the pilus backbone gene (*spy0116*) (Fig. 1A) in the background of M4 GAS strain 4063-05 to gain insight into its biological function. The mutation was verified by PCR analysis (Fig. S1B). Reduced surface pilus expression in the M4 GAS mutant (here designated Δ0116) was detected by flow cytometry analysis with rabbit antisera recognizing the T4 antigen (Fig. 1B), and restoration of pilus expression in the mutant was achieved by complementation with a plasmid vector carrying an intact copy of the *spy0116* gene (Fig. 1C). Anti-T4 antigen antibodies detected a high-molecular-weight covalent pilus in cell wall extracts from the wild type (WT), whereas no bands were observed in extracts from the Δ0116 mutant (Fig. 1D). In addition, characteristic fibrous structures protruding circumferentially from the bacterial surface clearly visualized in the WT were absent in the Δ0116 mutant (Fig. 1E). Immunogold staining with antisera recognizing the T4 antigen also revealed pilus structures extending from the WT surface but not the Δ0116 mutant surface (Fig. 1F), indicated that T4 antigen, encoded by *spy0116*, is indispensable for proper formation of intact pilus structures. Deletion of the pilus backbone T4 antigen did not attenuate bacterial growth, as the mutant and parental strains grew equally well in bacteriologic medium and RPMI-based culture medium used for the *in vitro* cell-based assays of the present study (Fig. S1C and D)

10.1128/mBio.01580-20.1 FIG S1 (A) Draft genome of M4 GAS strain 4063-05. (B) PCR validation to demonstrate replacement of the *spy0116* gene with the *cat* gene in the Δ0116 mutant. Comparison of the growth kinetics of M4 GAS WT and the Δ0116 strain in Todd-Hewitt broth medium (C), RPMI 1640 medium supplemented with 10% FBS (D), and 2% FBS (E). Download FIG S1, TIF file, 0.8 MB. Copyright © 2020 Chen et al. 2020 Chen et al. This content is distributed under the terms of the Creative Commons Attribution 4.0 International license.

### Roles of M4 GAS pili in biofilm formation and host cell adherence.

**FIG 2:**
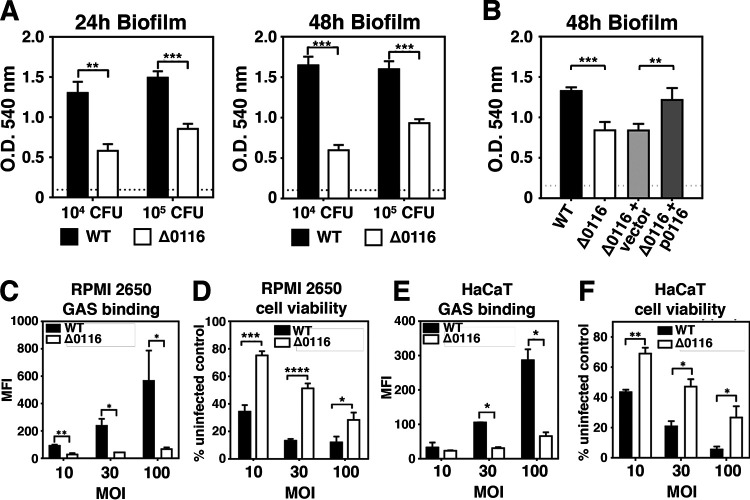
Roles of M4 GAS pilus in biofilm formation, cell adherence, and GAS-induced cell death. GAS was grown in 96-well polystyrene plates for 24 and 48 h. Biofilm mass of M4 GAS WT, Δ0116 mutants (A), and complemented strains (B) was stained with crystal violet and quantified by measuring the absorbance at 540 nm. Representative results from two independent experiments are shown as means ± SEMs. Flow cytometric analysis of GFP-expressing GAS adherence to RPMI 2650 (C) and HaCaT cells (E). Adherence was expressed as the mean fluorescent intensity (MFI) of the bacterium-associated cells (GFP^+^). GAS-induced cell death was assessed by crystal violet staining of surviving RPMI 2650 (D) and HaCaT cells (F) upon GAS infection. Data shown are means ± SEMs pooled from two independent experiments. Statistical significance was determined by unpaired *t* tests. *, *P* < 0.05; **, *P* < 0.01; ***, *P* < 0.001; ****, *P* < 0.0001.

To elucidate virulence-associated functions of M4 GAS pili, we first analyzed their roles in GAS biofilm formation and host cell adherence. WT M4 GAS produced strong biofilms on polystyrene plates, whereas the nonpiliated Δ0116 mutant showed severe reductions in biofilm formation at both 24 h and 48 h (Fig. 2A). Restoration of surface pilus expression in the *trans*-complementation strain (Fig. 1C, Δ0116+p0116) rescued biofilm formation capability to WT levels (Fig. 2B), corroborating key roles of the *spy0116* gene product in pilus structure and related biological functions. WT and Δ0116 mutant GAS were next compared for their adherence phenotypes on human nasal septum epithelial cells (RPMI 2650) and keratinocytes (HaCaT), relevant to primary GAS colonization of respiratory and skin epithelia. For these experiments, each strain was transformed with a green fluorescent protein (GFP) expression construct, incubated with the cells for 30 min, and then cells with bound bacteria (GFP^+^) were quantified by flow cytometry. WT GAS showed dose-dependent binding to RPMI 2650 cells and HaCaT cells, whereas significantly reduced binding was observed at each multiplicity of infection (MOI) for the isogenic Δ0116 mutant (Fig. 2C and D). Moreover, robust pilus-mediated cell adherence contributed to the significant cell death of WT-infected RPMI 2650 (Fig. 2E) and HaCaT cells (Fig. 2F), whereas marked reductions in cytotoxicity at all the tested MOIs were seen with the Δ0116 mutant. Together, our results indicate that surface display of FCT-5 pili in M4 GAS contributes to biofilm formation and cell adherence.

### M4 GAS pili play a crucial role in murine pathogenicity.

**FIG 3:**
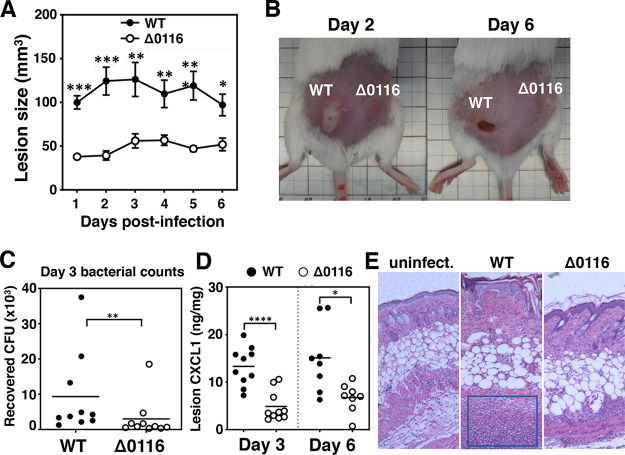
Pilus expression promoted M4 GAS persistence in a subcutaneous infection model. (A) Lesion size development was recorded over a 6-day time course. Representative results from two independent experiments are shown as means ± SEMs. (B) Representative photographs of necrotic skin lesions from infected mice. Bacterial counts (C) and CXCL1 production (D) in the infected skin are shown as means ± SEMs. (E) Representative hematoxylin and eosin staining of uninfected and infected skin sections. Differences between WT and Δ0116 mutant infected groups (*n* = 8 to 10) were calculated by Mann-Whitney *U* test (A, C, and D). *, *P* < 0.05; **, *P* < 0.01; ***, *P* < 0.001, ****, *P* < 0.0001.

The contribution of FCT-5 pili to M4 GAS skin infection was examined in a murine subcutaneous infection model, in which the pathogen produces necrotizing lesions or ulcers resembling human necrotizing fasciitis. Animals were infected with either WT or Δ0116 mutant GAS in opposing ﬂanks, and lesion sizes were monitored over a 6-day time course. Mice infected with WT GAS developed purulent skin lesions beginning day 1 postinfection, with lesion sizes peaking at day 3 (Fig. 3A). Infection with the Δ0116 mutant resulted in significantly smaller lesions at every time point postinfection than infection with WT strain throughout the observation period (Fig. 3A and B). Consistent with the larger lesion sizes caused by WT GAS, more bacterial CFU were recovered from the WT-infected lesions collected at day 3 (Fig. 3C), which is associated with higher levels of the neutrophil chemokine CXCL1 in the lesions (Fig. 3D). Hematoxylin and eosin (H&E) staining of skin infected with the Δ0116 mutant revealed a relatively intact dermal surface and deeper dermis with limited infiltration of immune cells compared to the marked inflammatory necrosis (indicated by the blue box) seen in WT-infected skin (Fig. 3E).

**FIG 4:**
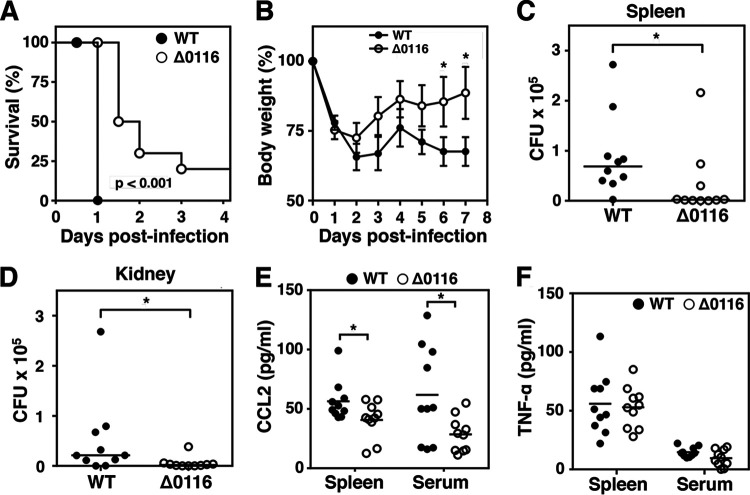
Pilus expression was necessary for M4 GAS virulence in a systemic infection model. Kaplan-Meyer survival curves (A) and body weight measures (B) of mice infected intraperitoneally with 9 × 10^8^ CFU and 3 × 10^8^ GAS, respectively. Bacterial loads in spleens (C) and kidneys (D) were determined 18 h postinfection in mice infected with 1 × 10^8^ GAS. CCL2 (E) and TNF-α (F) production in spleens and sera was determined 18 h postinfection in mice infected with 10^8^ GAS. Differences between WT and Δ0116 mutant infected groups (*n* = 10) were calculated by Mann-Whitney *U* test (B to F). *, *P* < 0.05.

Targeted mutagenesis studies suggest a serotype-dependent role of pili in GAS virulence, wherein expression of pili attenuates virulence in M1 (FCT-2) and M49 (FCT-3) GAS backgrounds (22, 25), whereas pili are required for M53 (FCT-3) and M2 (FCT-6) GAS to cause invasive infection (23, 26). We extended our analysis of the contribution of pili to M4 GAS invasive disease in a systemic (intraperitoneal) infection model. Mice challenged with the Δ0116 mutant had significantly less mortality over time than mice infected with the WT parent strain, where all animals rapidly succumbed within 24 h postinfection at the lethal dose of 9 × 10^8^ CFU/mouse (Fig. 4A). When infecting with one-third of the lethal dose (3 × 10^8^ CFU/mouse), WT-infected mice showed aggravated weight loss compared to that of the Δ0116 mutant-infected animals over a 7-day monitoring period (Fig. 4B). Moreover, the bacterial loads in the spleens and kidneys of WT-infected mice were 10-fold higher than those from animals challenged with the Δ0116 mutant (Fig. 4C and D). Elevated expression of CCL2, a chemokine involved in macrophage recruitment and polarization during inflammation, was observed both in the spleen homogenates and sera collected from WT-infected mice compared to those from the Δ0116 mutant-infected mice (Fig. 4E), whereas the levels of proinflammatory cytokine tumor necrosis factor alpha (TNF-α) (Fig. 4F) were comparable in the two groups. Thus, our data suggest that pilus expression contributes to the systemic virulence of nonencapsulated M4 GAS in addition to its roles in colonization and localized necrotic skin infection phenotypes.

### Pilus expression confers M4 GAS bloodstream survival.

**FIG 5:**
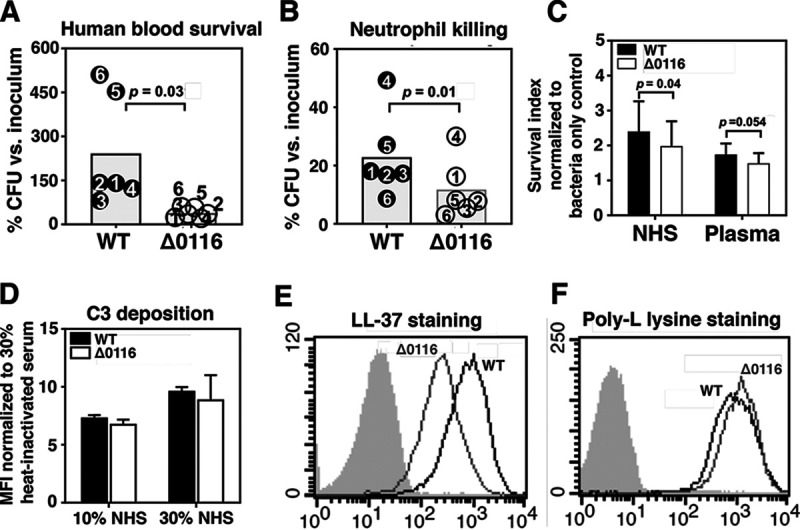
Pilus expression promoted M4 GAS survival in whole blood, neutrophils, and serum. Survival of M4 WT and the Δ0116 mutant in heparinized human blood (A) and human neutrophils (B). The survival rates were calculated by comparing the recovered CFU after 2 h of incubation to the initial inoculum. Experiments were performed with blood or neutrophils collected from different healthy donors. (C) Survival of M4 WT and the Δ0116 mutant in 5% normal human serum (NHS) and 5% plasma was quantified after 2 h of incubation at 37°C. Pooled data from 4 independent experiments are shown as means ± SEMs. (D) C3b deposition on M4 WT and the Δ0116 mutant in the presence of 10% and 30% NHS. Analysis of LL-37 (E) and poly-l-lysine (F) binding to M4 GAS and the Δ0116 mutant by flow cytometry.

To examine if pilus expression protects M4 GAS against immune clearance in human blood, we compared survival of WT and Δ0116 M4 GAS in freshly isolated human whole blood from different individuals. Consistent with the attenuated virulence in experimental animals, the pilus-deficient Δ0116 mutant was attenuated in replication in human whole blood compared to that of the WT strain (Fig. 5A). Immune components potentially contributing to pilus-dependent enhanced blood survival of M4 GAS were further explored by comparing the bactericidal effects of neutrophils, serum proteins, and cathelicidin antimicrobial peptides (AMPs) on WT versus Δ0116 mutant strains. Significantly more WT than nonpiliated Δ0116 mutant M4 GAS were recovered when cocultured with purified human neutrophils (Fig. 5B). WT GAS also survived slightly better than the Δ0116 mutant in 5% normal human serum (NHS), with a nonsignificant trend (*P* = 0.054) for survival in 5% plasma (Fig. 5C). These findings appeared independent of complement activity, as comparable C3b deposition was detected on the surfaces of the WT and Δ0116 mutant strains (Fig. 5D). Conversely, reduced binding of 6-carboxyfluorescein (FAM)-labeled LL-37 (Fig. 5E) and reduced sensitivity to human (LL-37) and murine (CRAMP) cathelicidin killing (MICs in Table 1) were observed in the pilus-deficient Δ0116 mutant. Diminished binding of the cationic AMP LL-37 was likely not due to a change in surface charge of the Δ0116 mutant after loss of pilus expression, since the WT and mutant strains showed equivalent binding to cationic poly-l-lysine compounds (Fig. 5F). Together, our data indicate that M4 GAS pili aid in resistance to neutrophil killing mechanisms; however, increased binding and sensitivity to host cathelicidin AMPs is mediated by the same surface structure.

**TABLE 1:** MIC of cathelicidins against M4 GAS

Cathelicidin	MIC (μM)		
	M4 WT	M4 WT+Hp	M4 Δ0116
LL-37	2	8	16–32
CRAMP	2	8	8–16

### M4 GAS binding of haptoglobin subverts immune defense mechanisms.

**FIG 6:**
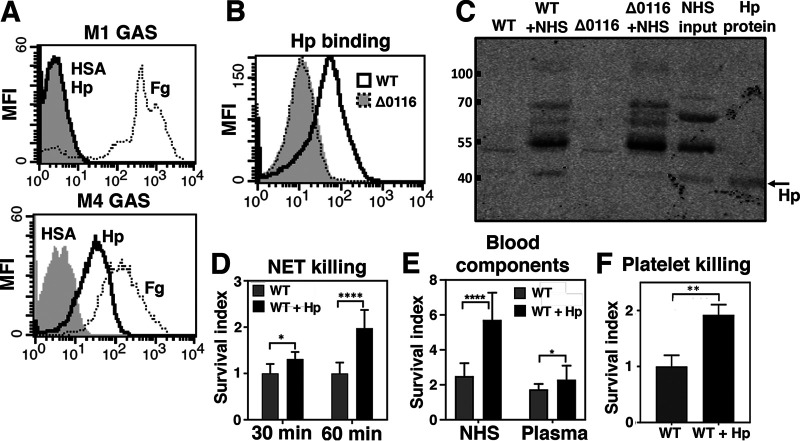
Acquisition of haptoglobin conferred M4 GAS the resistance to host innate immune defenses. (A) M1 and M4 GAS were stained with 2 μg of DyLight 488-labeled human serum albumin (HSA; gray fill), haptoglobin (Hp; black line), and fibrinogen (Fg; black dotted line) followed by flow cytometric analysis. (B) Deletion of *spy0116* diminished Hp binding. Gray fill, WT and Δ0116 strains stained with HSA; black line, M4 WT stained with Hp; gray dotted line, Δ0116 mutant stained with Hp. (C) WT and Δ0116 GASs were incubated with 50% human serum and washed thoroughly, and then the precipitates were probed with anti-Hp antibodies to detect the presence of Hp. (D) Survival of M4 WT and Δ0116 strains in PMA-induced neutrophil extracellular traps (NET) (D), 5% normal human serum (NHS) and plasma (E), and thrombin-activated platelets (F) was quantified 2 h after incubation at 37°C. Experiments were performed in triplicates, and pooled data from 2 to 4 independent experiments are shown as means ± SEMs. Statistical significance was determined by unpaired *t* tests. **, *P* < 0.01; ****, *P* < 0.0001.

In contrast to findings reported for GAS M1T1 pili which did not alter the bacterium’s susceptibility to cathelicidins (22), piliated WT M4 GAS showed increased binding and susceptibility to cathelicidins (Fig. 5E and Table 1), seemingly in contrast to the superior replicating capability of WT M4 GAS in human blood (Fig. 5A). A unique interaction between Hp, an immune-suppressive serum protein, and the T4 antigen of M4 GAS was long ago demonstrated but not ascribed a biological function (34, 35). We tested whether sequestration of Hp by surface pili might protect nonencapsulated M4 GAS against cathelicidin AMP killing and further enhance its resistance to host innate immune clearance and promote human blood survival. We stained GAS with DyLight 488-labeled Hp followed by flow cytometry analysis to measure interaction with Hp. Fibrinogen (Fg), known to interact with various GAS serotypes, was labeled in the same manner as a control. Whereas Fg showed equivalent binding to M4 and M1 GAS, Hp binding was distinctively seen in M4 GAS but not in M1 GAS or in the pilus-deficient Δ0116 mutant (Fig. 6A and B). In addition, WT but not Δ0116 M4 GAS directly pulled down endogenous Hp from pooled human sera (Fig. 6C), further supporting the key role of T4 antigen in Hp interaction. Hp is one of the most abundant acute-phase proteins (APPs) in serum, with concentrations ranging from 0.5 to 3 mg/ml (41). To test whether interaction with Hp confers a survival advantage to M4 GAS when encountering host defense machineries, WT M4 GAS was incubated with purified Hp (1 mg/ml) for 15 min and then washed with PBS to remove unbound Hp before experimental analysis. Survival of WT GAS and Hp-coated GAS was compared in the presence of AMPs and neutrophil extracellular traps (NET) in which cathelicidins are abundant (42). Coating M4 GAS with Hp not only reduced the susceptibility to LL-37 and CRAMP (Table 1) but also promoted the resistance to NET-mediated killing (Fig. 6D). In addition, acquisition of Hp by WT M4 GAS generated a profound surviving advantage over naked M4 GAS in human serum but only a modest statistically significant survival benefit in plasma (Fig. 6E). This contrast suggested that platelet-derived antimicrobial peptides released after blood coagulation may be a factor in Hp-mediated resistance to immune clearance. Indeed, acquisition of surface Hp protected GAS from the thrombin-activated platelet killing (Fig. 6F). Together our results indicate that T4 antigen of M4 GAS plays an important role in detoxifying the bactericidal activity of antimicrobial substances released from activated neutrophils and platelets, in part via sequestration of host Hp on the bacterial surface.

## DISCUSSION

GAS isolates rich in HA capsule are extremely virulent and positively associated with deeply invasive infections (6, 7, 43). Mimicking a common component of the human extracellular matrix, HA capsule contributes to GAS pathogenicity in many ways, such as forming a protective physical barrier, facilitating host epithelial cell interactions, and protecting GAS from immune surveillance and phagocytic killing (5–8). Intriguingly, a sustained increase in reported infections caused by capsule-negative GAS has been observed in recent epidemiologic surveillance (9–14). In this study, we show that the T4 antigen, corresponding to pilus backbone protein encoded by the *spy0116* gene, contributes to virulence properties of M4 GAS that lacks the entire *hasABC* operon required for HA capsule biosynthesis. Deletion of *spy0116* abolished pilus polymerization in M4 GAS, and Δ0116 mutants exhibited markedly reduced biofilm formation, epithelial cell binding, and cytotoxicity to target cells. Pilus expression contributes to M4 GAS survival in human blood and disease pathogenesis in murine models of necrotizing skin infection and systemic disease. A unique interaction between T4 antigen and Hp, a major acute-phase protein produced upon infection, further protects M4 GAS from neutrophil and antimicrobial peptide (AMP)-mediated killing. Our data suggest that the M4 GAS pilus may contribute to initial colonization and promote systemic virulence, in part through its interaction with Hp to blunt host innate defenses.

T antigens, classically employed for supplementary GAS serological typing, were first discovered by Rebecca Lancefield in the 1950s and are now known to represent the backbone protein that polymerizes to compose the GAS filamentous pili (28, 30). Encoded within the FCT region, the role for pilus in GAS virulence has been examined in FCT-2, -3, -4, and -6 types, but pilus encoded within FCT-5 region in nonencapsulated M4 strains remains uncharacterized for its regulation, function, and contribution in virulence. Electron microscopy and immunoblot results presented here show that deletion of the *spy0116* gene specifically abolishes the surface T4 antigen and fibrous pilus expression, without affecting the expression of the most abundant M4 surface proteins (Fig. 1). In addition, deletion of *spy0116* significantly reduced biofilm formation and bacterial adherence and cytotoxicity to RPMI 2650 upper respiratory epithelial cells and HaCaT keratinocytes (Fig. 2). Our observations are in line with previously reported epithelial adherence properties of GAS pili from other FCT types (21, 24–26, 44).

Nine different GAS FCT types have been identified and show high heterogeneity in gene content, gene order, and gene sequence, suggesting significant functional diversity of pili in this bacterial species (45). Loss of pilus expression in serotype M1 (FCT-2) and M3 (FCT-3) GAS resulted in a more invasive phenotype with increased human blood survival and/or increased virulence in an invasive mouse infection model (22, 46). In contrast, pilus expression in M2 (FCT-6), M49 (FCT-3), or M53 (FCT-3) GAS was required for the full disease-causing potential of each strain (25, 26, 47, 48). Increased mRNA expression of pilus backbone (bp1) caused by inactivation of the CovRS two-component transcriptional regulation system has been associated with increased virulence of a nonencapsulated M4 GAS strain in a murine intraperitoneal challenge model (49). We also observed a significant contribution of M4 pili in the invasiveness of M4 GAS in murine subcutaneous skin infection and systemic intraperitoneal infection (Fig. 3 and 4), which points out the potential contributions of pili to thwarting the host immune defenses and helping to compensate for the absence of HA capsule in M4 GAS.

In line with the role of M4 pili in promoting *in vivo* virulence in animal models, piliated M4 WT also displayed enhanced survival in human whole blood *ex vivo* compared with that of the Δ0116 mutant (Fig. 5A). Consistent with these observations, pilus expression protected M4 WT from neutrophil killing (Fig. 5B). Contrasting contributions of pili to the sensitivity to cathelicidin AMPs have been observed in different Gram-positive bacteria, in that pili of serotype V group B *Streptococcus* (GBS) intercept LL-37 to protect the bacterium from killing, whereas pili do not influence LL-37 sensitivity in M1T1 GAS and serotype III GBS (22, 50, 51). Here, in the case of M4 GAS, we found that pilus expression increases the susceptibility to human LL-37 and murine CRAMP (Table 1). Initial binding to the anionic bacterial surface is required for cationic AMPs to exert their antimicrobial activity, and reducing the negative electrical charge of bacterial surface is a common strategy to increase bacterial AMP resistance (52, 53). Elimination of T4 antigen in M4 GAS did not affect its surface electrostatic charge, but specifically diminished its association with LL-37 (Fig. 5E and F). Our data suggest that M4 GAS pili, instead of intercepting AMPs for protection, more likely represent a surface-expressed target of AMPs that consequently increases susceptibility in the absence of the protective HA capsule. This higher AMP susceptibility observed in the piliated WT strains may be an evolutionary trade-off paid for the essential role of the pilus in biofilm formation or host epithelial cell interactions in colonization.

Cloaking or mimicking host molecules to facilitate host interaction, mask from immune surveillance, resist phagocytosis, and promote intracellular survival has been reported in several bacterial pathogens (54, 55). GAS interacts with various host serum factors, including fibrinogen, fibronectin, immunoglobulins, plasminogen, factor H, and C4BP, and this binding activity contributes to many steps in colonization and infection processes (32, 33). Of note, several GAS-interacting serum factors are acute-phase proteins (APPs) that are markedly upregulated during inflammation or in response to infection (41). This pattern of APP regulation suggests a role in modulating the host immune homeostasis and antimicrobial defense machinery. Haptoglobin (Hp) is one of the most abundant APPs, second only to albumin and immunoglobins, and its serum levels can be upregulated up to 8-fold in response to infection (56). Our data demonstrate that Hp preferentially interacts with M4 GAS (but not M1 GAS) and this interaction is mediated by T4 antigen, since the Δ0116 mutant loses Hp-binding capability (Fig. 6A to C).

Hp was proposed to act as an antimicrobial protein based on its ability to complex with hemoglobin released during hemolysis and limit the heme iron availability to invading pathogens (57). Our results contrast with the proposed antimicrobial role of Hp by showing that WT M4 surface sequestration of Hp significantly reduced the bacterium’s sensitivity to LL-37 and CRAMP (Table 1) and killing in neutrophil extracellular traps in which LL-37 is abundant (Fig. 6D). Indeed, the role of Hp-hemoglobin complexes in host-pathogen interactions is more complicated than previously imagined, since instead of deprivation of iron, several bacterial pathogens, including Haemophilus influenzae, Neisseria meningitidis, and Staphylococcus aureus, acquire iron through sequestering Hp-hemoglobin complexes via surface receptors, (58–60). In addition to reducing the susceptibility of M4 WT to cathelicidins, Hp sequestration significantly enhanced the M4 WT survival upon exposure to thrombin-activated platelets and human serum that contains high levels of platelet-derived antimicrobial peptides released during the clotting process (Fig. 6E and F). Binding of the highly abundant Hp may protect M4 GAS from AMP killing by increasing steric hindrance on the bacterial surface, especially as Hp is upregulated in the acute-phase response to infection.

In summary, our results reveal that the M4 GAS pilus contributes to GAS virulence phenotypes that may aid in initial colonization and simultaneously promotes systemic virulence by co-opting the biology of Hp to mitigate host antimicrobial defenses.

## MATERIALS AND METHODS

### Bacterial strains and mutant construction.

GAS strain 4063-05 (*emm*4, T-type 4) was originally isolated from the blood of a patient in Georgia, USA, in 2005 and cultivated in THY (Todd-Hewitt broth [Acumedia] containing 2% yeast extract [BD]). A precise in-frame allelic replacement of the pilus backbone *spy0116* gene with the chloramphenicol acetyltransferase (*cat*) gene was generated in strain 4063-05 using a previously described method (61) with primers listed in Table 2. Briefly, DNA fragments (∼900 bp) directly upstream and downstream of *spy0116* were individually PCR amplified from chromosomal DNA using primers with 19-bp extensions matching the 5′ and 3′ ends of the *cat* gene. The flanking sequence PCR products were then joined with the *cat* gene by fusion PCR, and the resulting amplicon was cloned into temperature-sensitive suicide erythromycin resistance vector pHY304 to generate the knockout vector, pHY-spy0116. This vector was transformed into GAS 4063-05 by electroporation, and single recombination events were identified at 37°C under 5 μg/ml erythromycin selection. Selection was relaxed by serial passage at 30°C without antibiotics, and the occurrence of double-crossover events was identified as loss of erythromycin resistance. The replacement of the target gene by *cat* was verified by PCR using appropriate primers listed in Table 2. For complementation, the full-length *spy0116* gene with a 1,000-bp upstream fragment was amplified from GAS strain 4063-05 by primers listed in Table 2, and PCR products were cloned into the pLZ12Km2-P23R-TA plasmid (gift from Thomas Proft, University of Auckland) carrying the kanamycin resistance gene, creating pLZ-*spy0116* (here designated p0116). The plasmid was electroporated into the Δ0116 mutant to generate complemented strain Δ0116+p0116, which was cultured in THY plus 200 μg/ml kanamycin to maintain the plasmid. Bacteria were grown to mid-log phase for experiments except where indicated.

**TABLE 2:** Primers used in this study

PCR product	Primers (5′→3′)
Up region of *spy0116*	CGACTCGAGCAAAGTCAACTAAAGATACGGGG
	CAGTGATTTTTTTCTCCATCTGTTCTCTCCTATTTGTAAAAAAT
Dn region of *spy0116*	GTGGCAGGGCGGGGCGTAATTTGTTAGAAAAATGGGAGG
	CGAGGATCC-GCAAAACCAAGTAAAACCACC
Chloramphenicol resistance *cat* gene	ATGGAGAAAAAAATCACTGGATATACC
	TTACGCCCCGCCCTGCCACTCATCGCA
*spy0116* flanking region	CAGGGGATAGTAACGTCGACG
	GATTTAAACGATGCCGTTTG
*spy0116* complementation	CCGCGG-GGGGATAGTAACGTCGACGGAGCC
	AGATCTCTATTCCGATTTATCATTTTTATTAC

### Whole-genome sequencing and MLST analysis.

Genomic DNA was extracted from M4 GAS strain 4063-05 and subjected to a Pacific BioSciences (PacBio) Sequel sequencing system to obtain long-read sequences by Genomics (New Taipei City, Taiwan). The finished genome was assembled with Flye v2.7 software. Multilocus sequence typing (MLST) analysis was performed to determine the sequence type (ST) of M4 GAS strain 4063-05 used in this study by analyzing the sequences of seven housekeeping genes as previously described (62). Strain 4063-05 belongs to ST39 as assigned by the MLST website (https://pubmlst.org/spyogenes/).

### Cell culture.

RPMI 2650 (ATCC CCL30) and HaCaT cells (63) were maintained in RPMI 1640 and Dulbecco’s modified Eagle medium (DMEM) supplemented with 10% fetal bovine serum (FBS), respectively. Neutrophils were isolated from healthy donors (with use and procedures approved by the National Taiwan University [IRB 201711091RIND]) using a PolyMorphPrep kit (Axis-Shield, Oslo, Norway) per the manufacturer’s instructions, while platelets were prepared by centrifugation from blood anticoagulated with citrate-dextrose solution (Sigma) as previously described (64).

### Detection of pilus expression by flow cytometry, transmission electron microscopy, and immunogold electron microscopy.

For flow cytometry analysis, mid-log-phase and stationary-phase GAS were stained with rabbit antisera against the GAS T4 antigen (Abcam) followed by Alexa Fluor 647-conjugated secondary antibodies (BioLegend). The stained samples were analyzed by FACSCalibur with CellQuest software. For transmission electron microscopy, mid-log-phase GAS were fixed with 0.1 M sodium cacodylate buffer containing 4% paraformaldehyde and 2.5% glutaraldehyde at room temperature for 2 h. The fixed bacteria were washed, resuspended with phosphate-buffered saline (PBS), dropped to a grid (TAAB), settled for 5 min, and air-dried. Cells were visualized with an H-7100 transmission electron microscope (Hitachi) at an accelerating voltage of 100 kV after staining with 1% phosphotungstic acid. For immunogold electron microscopy, mid-log-phase GAS were washed, resuspended with PBS, dropped to carbon-coated Formvar-covered nickel grids (TAAB), and settled for 5 min. Bacteria on the grids were then fixed with 2% paraformaldehyde for 5 min at room temperature and incubated with 1% bovine serum albumin (BSA)-PBS for 30 min at room temperature. Pili were labeled with rabbit antisera recognizing GAS T4 antigen at room temperature for 30 min followed by 18-nm gold-conjugated secondary antibodies (Jackson ImmunoResearch) and observed with an H-7100 transmission electron microscope.

### Cell wall extraction and Western blot analysis.

Cell wall extracts of GAS were prepared using mutanolysin as described previously (28). Briefly, overnight grown GAS were washed with PBS, resuspended in the protoplast buffer (0.1 M KPO_4_ [pH 6.2], 10 mM MgCl_2_, 40% sucrose, 2 mg/ml lysozyme, 400 U mutanolysin [Sigma], and EDTA-free protease inhibitor [Roche]), and then incubated at 37°C with constant rotation for 3 h. Supernatants containing surface proteins were collected, separated on 4% to 15% SDS-PAGE gels (Bio-Rad), transferred to a polyvinylidene difluoride (PVDF) membrane, detected with primary rabbit antisera recognizing GAS T4 antigen and IRDye 800CW-conjugated secondary antibodies (LI-COR), and visualized and quantified with a LI-COR Odyssey scanner and software.

### Cell adherence and survival assays.

Human nasal septum (RPMI 2650, 1 × 10^6^/well) and keratinocyte (HaCaT, 2 × 10^5^/well) cells were plated in a 24-well plate 1 day prior to the assay. Mid-log-phase GAS with transformed plasmids encoding GFP were thoroughly mixed to disrupt potential aggregation and added to the cells at multiplicities of infection (MOIs) of 10, 30, and 100. Plates were centrifuged for 5 min at 1,600 rpm to initiate bacterial contact and incubated for 30 min at 37°C. Infected cells were washed six times with PBS, detached with 5 mM EDTA-PBS, and analyzed by flow cytometry to quantitate the percentage of cells with bacterial association (GFP^+^). To measure the bacterial cytotoxicity to the infected cells, RPMI 2650 (2 × 10^5^/well) and HaCaT (2.5 × 10^4^/well) cells were plated in a 96-well plate 1 day prior to the experiment, infected with GAS at MOIs of 10, 30, and 100 for 1 h, followed by addition of penicillin and gentamicin to 10 and 100 μg/ml, respectively, and then cultured for an additional 23 h. After infection, cells were washed, fixed with methanol, and incubated with 0.2% crystal violet at room temperature for 10 min to stain viable cells. After extensive PBS washes, the bound dye was recovered with 100 μl of 1% sodium dodecyl sulfate (SDS). The cell survival index was quantitated by normalization of the optical density at 540 nm (OD_540_) of the infected cells to that of the uninfected control.

### Biofilm assay.

Mid-log-phase GAS were adjusted to 10^4^ CFU/ml and 10^5^ CFU/ml with C medium (0.5% proteose peptone no. 3, 1.5% yeast extract, 10 mM K_2_HPO_4_, 0.4 mM MgSO4, 17 mM NaCl) plus 30 mM glucose, seeded into a 96-well plate, and incubated at 37°C for 24 and 48 h, respectively. The culture medium was decanted, and the plates were washed three times with PBS and fixed with methanol. Adherent bacteria were stained with 0.2% crystal violet at room temperature for 10 min. After extensive PBS washes, the bound dye was extracted with 100 μl of 1% SDS. Biofilm formation was quantified by measuring the absorbance of the solution at 540 nm by a spectrophotometer.

### Mouse infection models.

All mouse experiments were conducted under a protocol approved by National Taiwan University college of Medicine Animal Care and Use Committee (IACUC 2016041). For soft tissue infection, mice (ICR, female, 6 weeks old, *n* = 8 to 10) were subcutaneously injected with M4 WT and Δ0116 strains (2 × 10^8^ CFU/mouse) in the shaved right and left back flanks, respectively. Lesion sizes were recorded by a camera with a fixed height and calculated by ImageJ software. Lesions were excised 3 days postinfection and homogenized in PBS with 1-mm zirconium beads (BioSpec) to analyze the bacterial burden and local cytokine production. For systemic infection, cohorts of 8 to 10 (female 8-week-old ICR) mice were intraperitoneally inoculated with 9 × 10^8^ CFU (for survival) or 3 × 10^8^ CFU GAS (for weight and bacterial load measurements) resuspended in 200 μl of 5% mucin-PBS (Sigma). Μouse weight and survival was monitored daily for 1 week. Disseminated bacteria were enumerated from spleens and kidneys collected from infected animals 20 h postinfection.

### Human whole blood, serum, and plasma killing assays.

For human whole-blood killing assays, 10^4^ CFU of mid-log-phase GAS were incubated with 450 μl of heparinized whole blood collected from healthy volunteers with constant rotation for 2 h. Surviving bacteria were enumerated by plating in triplicates on Todd-Hewitt agar (THA) plates. Bacterial survival index was calculated by dividing the number of surviving CFU by the initial bacterial inoculum. For serum and plasma bactericidal assays, 10^4^ CFU of mid-log-phase GAS were incubated with 5% normal human serum (NHS) or 5% plasma for 2 h, and surviving bacteria were enumerated by plating in triplicates on THA plates. Bacterial survival index was calculated by dividing the number of surviving CFU by the number of bacterial CFU recovered from untreated group.

### Neutrophil, neutrophil extracellular trap, and platelet killing assays.

For killing assays, human neutrophils (2 × 10^5^), NET from phorbol myristate acetate (PMA)-activated neutrophils (2 × 10^5^) and thrombin-activated platelets (10^7^) were inoculated with 10^5^ CFU of mid-log-phase GAS for 2 h. After incubation, NETs were treated with DNase I, and infected neutrophils and platelets were lysed by vigorously pipetting. The surviving bacteria were diluted and plated on THA plates for CFU enumeration. The bacterial survival index was calculated by dividing the number of surviving CFU by the initial bacterial inoculum.

### Susceptibility and binding to LL-37 and CRAMP.

To measure cathelicidin antimicrobial peptide sensitivity of GAS, mid-log-phase GAS, were adjusted to 1 × 10^5^ CFU/ml in DMEM plus 20% THY and incubated with various amount of human cathelicidin, LL-37 (AnaSpec), or murine cathelicidin, CRAMP (Bachem) for 24 h at 37°C. MIC was determined as the lowest concentration at which no growth was observed by absorbance at 600 nm. Assays were performed in triplicates, and where there was variation between replicates, the data are presented as a range. To examine bacterial surface charge and LL-37 interaction, mid-log-phase bacteria (5 × 10^7^) were stained with 5 μg of 5-FAM-LC-conjugated LL-37 (AnaSpec) and fluorescein isothiocyanate (FITC)-conjugated poly-l-lysine (Sigma), respectively, for 20 min at room temperature.

### Haptoglobin binding assay.

To determine the interaction between human serum protein and M4 GAS, mid-log-phase GAS (5 × 10^7^) were stained with 5 μg of the DyLight 488 (Thermo Fisher Scientific)-conjugated human Hp (MyBioSource), serum albumin (Sigma), or fibrinogen (Sigma) for 20 min at room temperature. The stained bacteria were washed and analyzed by FACSCalibur with CellQuest software. To test whether GAS can pulldown endogenous Hp from human serum, mid-log-phase GAS (2 × 10^8^) were incubated with 10% or 50% pooled human sera at 37°C for 15 min with rotation. The bacteria were washed twice with 1 ml PBS, and the bound proteins were eluted by boiling washed GAS in SDS-PAGE sampling buffer for 5 min. The eluted protein fraction was separated on SDS-PAGE, transferred to a PVDF membrane, detected with primary rabbit antisera recognizing human Hp (Genetex) and IRDye 800CW-conjugated secondary antibodies (LI-COR), and visualized and quantified with a LI-COR Odyssey scanner and software.

### Measurement of cytokines.

Cytokines in mouse lesions, spleen homogenates, and serums from infected animals were detected by corresponding commercial enzyme-linked immunosorbent assay (ELISA) kits (CXCL1 from R&D; TNF-α and CCL2 from eBioscience).

### Statistical analysis.

All statistical tests were performed using GraphPad Prism version 6 (GraphPad Software, Inc.). Experiments using human materials were performed with samples from a minimum of three different healthy volunteers. Data presented here were combined, normalized, and expressed as means ± standard errors of the means (SEMs) except where indicated. Mann-Whitney U test was used to compare the lesion size, cytokine secretion, and bacterial burden in tissues. A log rank test was used to compare the survival of animals. A two-tailed *t* test or one-way analysis of variance (ANOVA) with Tukey’s multiple-comparison test was used to compare the rest of data as indicated in the legends. A *P* value of <0.05 was considered significant for all tests.

### Data availability.

The complete genome sequence of M4 GAS strain 4063-05 has been submitted to GenBank under accession number CP051138.
