# Supplementary figures and images for: T4 Pili Promote Colonization and Immune Evasion Phenotypes of Nonencapsulated M4 Streptococcus pyogenes
Source: mBio. 2020 Jul 21;11(4):e01580-20. doi: 10.1128/mBio.01580-20 (PMC7374061; doi:10.1128/mBio.01580-20)

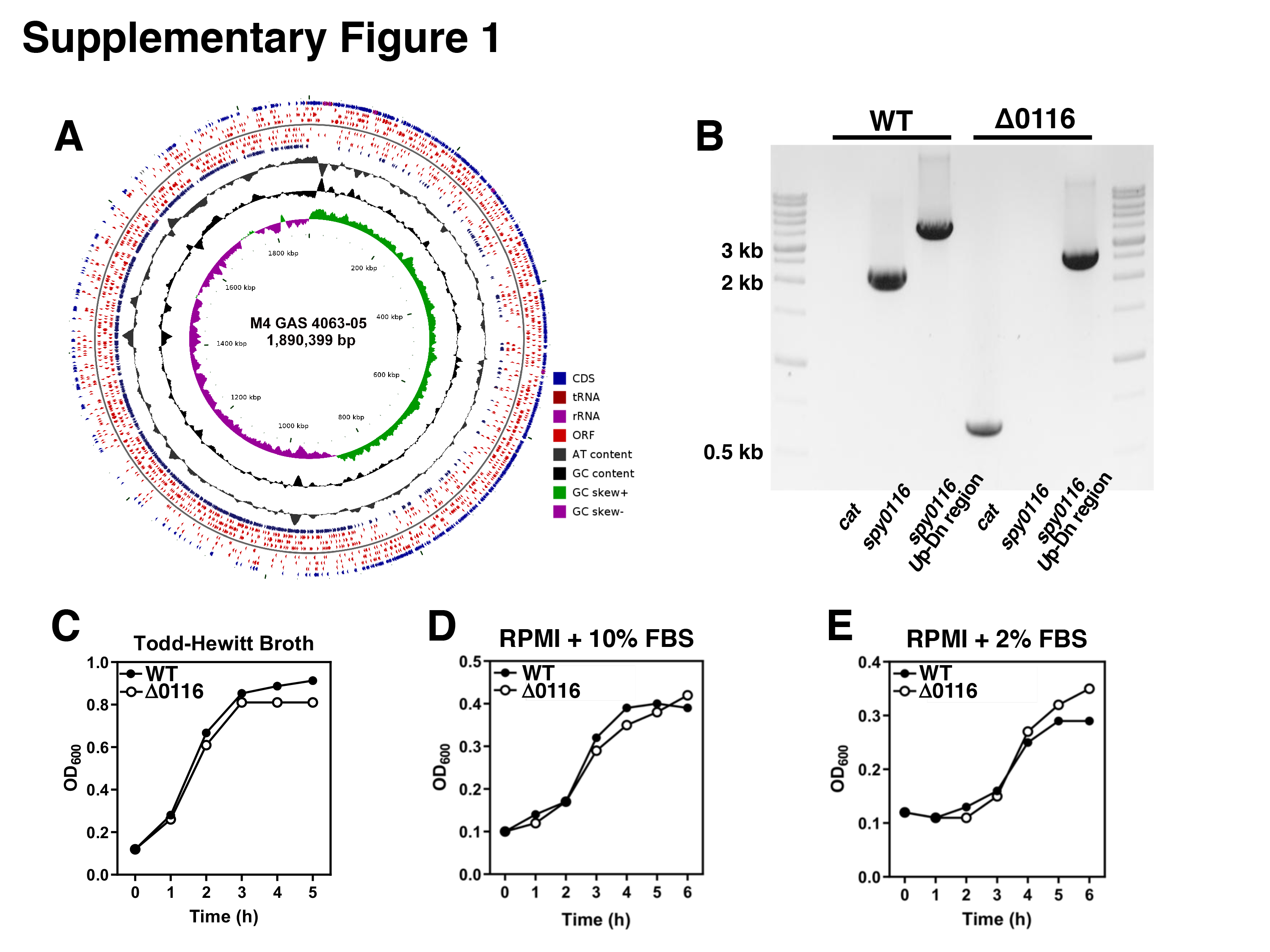

Supplement: FIG S1 [file mBio.01580-20-sf001.tif]
